# Tailoring intervention procedures to routine primary health care practice; an ethnographic process evaluation

**DOI:** 10.1186/1472-6963-7-125

**Published:** 2007-08-07

**Authors:** Yvonne JFM Jansen, Antoinette de Bont, Marleen Foets, Marc Bruijnzeels, Roland Bal

**Affiliations:** 1 Institute of Health Policy and Management, Erasmus MC Rotterdam, PO Box 1738, 3000 DR Rotterdam, The Netherlands; 2 Stichting Lijn 1 Haaglanden, PO Box 138, 2270 ACVoorburg, The Netherlands

## Abstract

**Background:**

Tailor-made approaches enable the uptake of interventions as they are seen as a way to overcome the incompatibility of general interventions with local knowledge about the organisation of routine medical practice and the relationship between the patients and the professionals in practice. Our case is the Quattro project which is a prevention programme for cardiovascular diseases in high-risk patients in primary health care centres in deprived neighbourhoods. This programme was implemented as a pragmatic trial and foresaw the importance of local knowledge in primary health care and internal, or locally made, guidelines. The aim of this paper is to show how this prevention programme, which could be tailored to routine care, was implemented in primary care.

**Methods:**

An ethnographic design was used for this study. We observed and interviewed the researchers and the practice nurses. All the research documents, observations and transcribed interviews were analysed thematically.

**Results:**

Our ethnographic process evaluation showed that the opportunity of tailoring intervention procedures to routine care in a pragmatic trial setting did not result in a well-organised and well-implemented prevention programme. In fact, the lack of standard protocols hindered the implementation of the intervention. Although it was not the purpose of this trial, a guideline was developed. Despite the fact that the developed guideline functioned as a tool, it did not result in the intervention being organised accordingly. However, the guideline did make tailoring the intervention possible. It provided the professionals with the key or the instructions needed to achieve organisational change and transform the existing interprofessional relations.

**Conclusion:**

As tailor-made approaches are developed to enable the uptake of interventions in routine practice, they are facilitated by the brokering of tools such as guidelines. In our study, guidelines facilitated organisational change and enabled the transformation of existing interprofessional relations, and thus made tailoring possible. The attractive flexibility of pragmatic trial design in taking account of local practice variations may often be overestimated.

## Background

The appropriateness of the randomised controlled trial (RCT) for evaluating complex interventions is heavily debated [1-3]. The Quattro project was such a complex intervention. It was a prevention programme for cardiovascular diseases (CVD) in high-risk patients in primary health care centres in deprived neighbourhoods. Multidisciplinary care teams (Quattro care-team) were to support high-risk patients on medicine use, smoking, diet and lifestyle changes. An RCT requires that a standardised intervention, with statistical measurable outcomes, be implemented uniformly within a homogenous target audience [1]. The notion of standardisation seems, however, to be at odds with more complex interventions that have to be implemented in complex health care settings [3]. While the outcomes of activities in real life practice may be highly dependent on the characteristics of the health care providers, the setting and the patients [1,2,4,5], trials of complex interventions are still considered to resemble the original intervention as much as possible, standardising both the content and delivery of the intervention [3].

In fact, the limited effectiveness of complex interventions is often attributed to the failure to tailor the design of interventions to local care practices [2]. The uptake of interventions is to be done by tailoring the intervention procedures to routine clinical decision-making and incorporating the heterogeneity of patients and health care professionals. It is argued that as an intervention is tailored to the unique characteristics of each practice, they may be more likely to become incorporated into the structure and function of daily operations resulting in sustainable effects [6]. This is confirmed by findings from studies on the diffusion and adoption of innovations in clinical practice. The adoption of innovations is often considered to take place according to linear models, but in practice innovations often have a dynamic and fluid nature resulting in their adoption – or parts thereof – over time [7].

To enable the uptake of Quattro care in routine practice, a tailor-made approach was used. Although the process for the intervention was fixed for participating general practices (i.e. four structured team meetings of the Quattro-care team and four individual patient education sessions per patient), local adaptations to the components and form were allowed to tailor the intervention to individual practice needs and organisation. This tailor-made approach – with the underlying intention to minimise intrusion into normal daily care – was meant as an opportunity for the participating health care centres to frame their preferred procedures for the intervention, instead of following external guidelines that might not be perfectly applicable to the specific context of these centres and which were seen as externally generated research interference. In this, the Quattro project followed the procedures of a pragmatic trial, which aims to measure the effectiveness of interventions under natural – non-experimental – conditions.

In this paper we are concerned with how the professionals tailored the Quattro intervention to their practice needs and organisation. So, as in the Quattro project multidisciplinary care teams had to provide the prevention of CVD by means of a stipulated process, the participating primary health care centres could tailor their components and form. Therefore our main questions for this ethnographic process evaluation of the Quattro project were: How did the health care professionals tailor the intervention in the participating health care centres? What role did the provided guideline have in tailoring the implementation of the intervention? Did the tailor-made approach have advantages for the implementation of the intervention?

### Quattro Care intervention

The Quattro project introduced and evaluated the effectiveness of multidisciplinary patient care teams for the prevention of cardiovascular diseases (CVD) in three primary health care centres located in deprived neighbourhoods of Rotterdam and The Hague. The core of the Quattro project was the collaboration between a practice nurse, a peer health educator, the GP, and assistant (hence Quattro care) in providing intensified preventive care. Multidisciplinary patient care teams are thought to improve the quality of care in general practice, as they are seen as a means of relieving the workload of GPs and to assist them in providing preventive activities [8-11]. GPs in deprived neighbourhoods have a great deal of information about patients, but – due to their high workload – do not use this information for case-finding and secondary prevention. They lack the time and the organisation to actively invite people for check-ups [9,11-13]. They do not actively assess risk-profiles either. According to the national recommendations, they should [14-18].

The intervention described the main tasks for the intervention team: GP (treatment task, overall medical responsibility), practice nurse (risk assessment, coordination and prevention tasks), assistant (logistic task) and peer health educator (ethnic specific health education)). Each intervention patient was to receive at least four individual patient education sessions provided by the practice nurse and/or peer health educator. At least four multidisciplinary team meetings were to be organised by the intervention team professionals to jointly establish treatment plans for intervention patients and monitor the patients' risk profiles based on the particular knowledge and abilities of each professional. Although the process for the intervention was fixed for participating general practices, adaptations to the components and form were allowed to tailor the intervention to the individual practice needs and organisation. The practice nurses were meant to be the coordinating axis of the rollout and implementation of the intervention in the practices. No additional guidelines were provided to support this organisation of activities. For the programme the health care professionals had to use the GP guidelines of the Dutch College of General Practitioners (NHG), for hypertension, hypercholesterolemia, diabetes mellitus, smoking and obesity.

For this programme patients were selected with a modifiable part of the absolute 10-year risk on cardiovascular diseases (CVD) of at least 20%, and were randomly assigned to three groups. Patients in the intervention group were to obtain Quattro-care and three-monthly assessments of their risk profile from the practice nurses. Patients from control group A were to receive usual GP care. The assistants were to perform the three-monthly risk assessments of this group, in order to prohibit contamination of the results of the aforementioned groups. Both the GPs and the patients were informed about the results of these assessments. It was thought to be ethically and practically unacceptable to assess a risk-profile and not to inform the patient and GP about the results. However, this approach of assessing risk and informing patients and GP would interfere with daily practice and could bias the results. Therefore, a blind control group B was needed to quantify the effect of the risk assessments. This group was to receive usual GP care and was measured once at the end of the study. The effectiveness of the multidisciplinary collaboration was assessed by comparing patients from the intervention group with those from control group A after one year follow-up with the outcome measure defined as the reduction achieved in the absolute 10-year risk of developing CVD. Control group B, aimed to quantify the effect of structured risk assessments performed in control group A, was compared with control group A. The follow-up period for the intervention and control group A was 12 months and the intervention programme lasted 9 months.

The pragmatic trial research team supported the participating primary health care centres in several ways. They assisted in finding compensation for this programme and the employment of the practice nurses. They organised an accredited course on organising effective multidisciplinary team meetings for the health care professionals before the start of the trial. Moreover, the research team assisted in case finding: they selected the high-risk patients from the centres' electronic patient records and were to receive patient monitoring data to calculate the effectiveness of this prevention project in routine medical practice. The study ran from August 2000 until December 2005.

## Methods

To evaluate feasibility, implementation and experiences with Quattro-care of health care professionals, patients and researchers, complementary qualitative research was considered necessary during the execution of the trial. Ethical approval for the Quattro Study, which also incorporated this complementary qualitative research, was obtained from the Health Ethics Board of Erasmus University Medical Center in Rotterdam. For our study we used an ethnographic design [19-21], as ethnographic process evaluations of the implementation of interventions and its adaptations in practice are necessary to be able to assess the validity and reliability of any effects of the interventions [22-24].

The first author observed four out of seven practice nurses in their daily work, each for five workdays each, and the multidisciplinary team meetings they attended from April 2003 till December 2004. Observing and interviewing the practice nurses was chosen as they had a coordinating key function in the intervention. During the observations no notes were made, because note taking was felt to intrude on the interactions between the practice nurses and their patients and colleagues. Written records were made immediately after leaving the health care centres.

From April 2003 till December 2004, the first author also observed the two researchers, the data manager, and an average of four research assistants in their work in the Quattro trial for two work days a week. The research progress meetings that were organised by the researchers, data manager, and the project leader were also observed. With the project leader, one of the researchers and the data manager audio taped semi-structured interviews were held, which were transcribed afterwards. Written records were made of all meetings, observations and conversations. Minutes of the meetings, research protocols and questionnaires used for the Quattro Study were also collected.

Throughout each observation it was possible to ask questions or to request clarification.

In addition, semi-structured interviews were held. With three practice nurses audio taped semi-structured interviews were held, and with two practice nurses non-audio taped semi-structured interviews were held due to objections to audio taping. In all interviews the practice nurses were asked about their experiences with the Quattro Study, their dealings with patients and their current activities in the primary health care centres. The interviews were transcribed immediately after the interviews. After the observation period, all transcripts, personal jot notes, minutes, and research documents were analysed more in-depth. We analysed all the information thematically through establishing overarching categories by manually identifying and coding all pieces of information without any predefined categories. By means of developing overarching categories (taxonomy) of emerging themes overall descriptions of our findings could be made in more general terms.

## Results

### Difficulties introducing Quattro care

As the management of participating primary health care centres volunteered to take part in the Quattro trial, they committed themselves to employing practice nurses for the implementation of the intervention in the practices (document practice invitation letter). The practice nurses had to negotiate with their colleagues to plan the implementation and to fine-tune the exact rollout of the Quattro project in practice.

The practice nurses experienced difficulties introducing Quattro care in their health care centres. As one of the formally interviewed practice nurses stated about this initial organisation of the Quattro programme: "It is assumed that practice nurses can apply the knowledge they learned during their education in an instant, organisational skills that is. This is not exactly the case. Before such a project is even organised it takes a lot of time deliberating and coming to agreements with your colleagues assigned to participate in this project on how the project should and could be organised internally" (interview practice nurse 07-06-2004).

First, it proved to be difficult for the peer health educators to gain the trust and confidence of the GPs. In one of the primary health care centres the practice nurse and peer health educator both agreed that the peer health educator would perform the same activities as the practice nurse, but only for the immigrant patients. But this was hard to bring about as the field note shows: "[...] *the practice nurse *writes a short version of the patient files on paper so the peer health educator can do her work. As *the first author asks *her why she does this she answers: "The peer health educators are not allowed to see the electronic patient records. Don't ask me the logic behind it, but the GPs decided the peer health educator will not have access to the system. [...] even though she has already worked here for five years" (field note 24-05-2004). So, although the peer health educators were to be seen as full members of the intervention team(s), the lack of status to negotiate their role in the intervention team disabled the peer health educators from becoming full members despite the enabling efforts of the practice nurses.

Second, the GPs were reluctant to take part: "The GPs consented to the project not really knowing what was being asked of them [...] that they too had to be involved in the treatment of patients and not just we as practice nurses, but when they finally realised this the multidisciplinary meetings were very hard to organise" (conversation practice nurse 12-05-2004). The GPs were not willing to alter their work schedules and activities to a great extent. "The practice nurses all were 'new' in the practices, but fairly soon it became obvious that the centres had not anticipated their practices would change" (conversation practice nurse 04-11-2004). Although the practice nurses were to be the coordinating axis of the implementation of the intervention, they were not able to discard the existing hierarchical positions of the GPs, peer health educators and assistants. In fact, it complicated the implementation of the intervention. As the project leader explained: "Quattro Care was set up to evaluate possible effects after its implementation in the health care centres. [...] It was the intention to provide the idea of Quattro Care to the health care centres and that they would take care of the organisational part themselves" (conversation project leader 10-09-2003). The practice nurses, however, were not able to implement and rollout the intervention in practice. "[...] It soon became clear to us that for the health care centres this was a problem" (conversation project leader 10-09-2003).

Thirdly, the decline in manpower and increasing workloads prevented the assistants of being part of the intervention team. As a result the practice nurses ended up seeing both intervention and control group patients. In two health care centres, the practice nurses were former assistants in these centres. When they became practice nurses, the number of assistants declined thus increasing their workload. To resolve this, the GPs used their hierarchical position to decide upon the issue at hand. "In the beginning the GPs thought of Quattro as a good idea. But when they saw it took more time doing intakes and counselling patients than they thought it would, they decided that the practice nurse would have to see both the intervention group and control group patients; the assistants already had too much work" (observation practice nurse 07-06-2004).

Thus, the existing interprofessional relations and local circumstances prohibited the intervention from being implemented as planned. A lot of effort had to be put into harmonising different insights and conflicting values. As all the centres employed multiple GPs and assistants – with their specialist medical interests – alongside only a minimal number of practice nurses and peer health educators, first basic organisational agreements had to be made about the formation of the intervention team and their deployment.

### Tailoring and standardisation: the Quattro Guideline

Confronted with these problems the researchers questioned whether they had to develop a guideline for the intervention to increase the fidelity to the intervention's key component: the multidisciplinary meetings. However, within the research team two different opinions prevailed about the researchers' role as interveners in the organisation of the centres. On the one hand, developing a guideline did not correspond with the tailor-made approach, as it meant externally generated research interference with the normal way of working. As the project leader explained: "In order to provide a 'good' implementation, *one of the researchers *developed a protocol based on the existing standards on hypertension, cholesterol, diabetes, etcetera. I opposed this at first [...] there must be as little as possible contact between the trial and the actual intervention" (conversation project leader 10-09-2003). Although research interference in daily care should be avoided for objectivity reasons, for the project leader this was not the main argument. For him, minimising research interference was about not forcing the health care professionals to use imposed (externally made) guidelines that do not fit the local organisational circumstances of these centres and about assessing "what trial based effects can be detected after such an implementation process in these practices" (conversation project leader 10-09-2003). So, according to the project leader, minimising research interference could give insight into what kind of organisational preconditions primary health care has to meet in order for the implementation of prevention projects to be successful in primary care.

On the other hand, for the researchers the development of a guideline implied a means of standardising the fidelity to the programme among the centres and a possibility of ensuring the protection of the outcome validity of their study. As one of the researchers indicated, she had the impression that the primary health care centres made a mess of things; they didn't register the rudimentary data, important for research (field note 03-02-2004). As it turned out, the different practice nurses had different ways of collecting the required data. For example, while attending one of the practice nurses' patient consultation, she told the first author that hip measuring was not done in the same manner among all practice nurses: "I noticed a few times that *my colleague *holds the measuring-tape more downwards instead of horizontally. The outcomes then will not be the same as in reality [...] the measurements then will differ a lot" (field note practice nurse 19-01-2004). So, as the patient monitoring data should be uniformly collected in order to be comparable, for the researchers the exactitude of the practice nurses' performance of medical-technical activities was considered a barrier for an uniform execution of the intervention among the participating health care centres. This is a problem that could, in theory, be resolved by providing them with a guideline. The tailoring we have described was, however, from the perspective of the pragmatic trial design not an intended process because it made establishing the effectiveness of the intervention by means of pre-established outcome measures difficult. (see [22] for more detail on the methodological dilemmas the researchers had to deal with when performing this pragmatic trial).

### Role of the guideline

After a long discussion about the different viewpoints the research team decided to develop a guideline. The Quattro guideline was foremost a medical-technical guideline developed by the research team members through combining the most recent separately existing GP-protocols for hypertension, hypercholesterolemia and diabetes mellitus type II, based on the national recommendation of the Dutch College of General Practitioners (NHG), with extra criteria for obesity and smoking.

The guideline described the procedural steps of the intervention's performance. It was written as a sequential procedure, that stated how and which medical-technical actions should be performed, what physical tests and measurements should be done and when, and which pharmaceutical treatments should be started depending on different measurement outcomes, but also how the physical examinations should be done and in what order. It also stated which patients had to be selected and on which grounds, who should perform the prevention, especially to whom, and what length the follow-up period should be in order to have sufficient data. But it also stated which data were necessary for calculating effectiveness outcomes and how and when this data should be returned to the trial researchers.

Furthermore, the guideline stated the elements of the prevention programme that had to be organised; at least four individual patient education sessions with intervention group patients and four multidisciplinary meetings with all intervention team members per patient. Moreover, it was indicated which topics should be discussed during these meetings and in which order, which professionals should attend these meetings, and what tasks the intervention team members were to have (document manual for the intervention). The guideline thus contained 'research' or 'evidence' elements as well as 'intervention' elements.

As the guideline provided contained instructions for the organisation of the multidisciplinary collaboration – though on a more general level, opposed to the more specified description of the medical-technical performance of the intervention – the instructions still remained open for the practice nurses' interpretation and adaptation to the local organisational circumstances and needs. Moreover, the guideline did not correspond with difficulties the practice nurses were presented with when organising the multidisciplinary collaborations among the different disciplines in the health care centres. Ergo, as the practice nurses were confronted with difficulties concerning the organisation of interprofessional cooperation and in that way achieve implementation of the new organisational structure of multidisciplinary patient care, they were provided with a guideline aimed at performing the medical-technical actions for the prevention of CVD, in which the implication of standardisation kept lingering in the background. The guideline did not enable the practice nurses to disregard all the existing hierarchical professional relations and/or local circumstances within the healthcare centres; i.e. the peer health educators and assistants were still not able to take part as members of the intervention teams. The practice nurses were for example still not able to arrange the multidisciplinary team meetings. And so these meetings were organised irregularly, often limited to only practice nurses and GPs, or were informal deliberations.

The guideline, however, did give the practice nurses the position they needed to negotiate the appropriateness of treatments with the GPs. Especially since within the centres different medical guidelines co-existed for dealing with cardiovascular diseases. In the health care centres the Quattro guideline came into use next to the (most) recent NHG recommendations focussing on hypertension, diabetes mellitus, and hypercholesterolemia separately. Although this mixed use in real life practice caused tensions between professionals, it did provide the practice nurses with the opportunity to negotiate the appropriateness of treatments. "[...] the *(Quattro) *guideline does have strict norm levels you have to adhere to. However, the GP doesn't use the same guideline. So, when a patient has a cholesterol level of 6 or 6.4; 6.5 *mmo/l*......ideally it has to be below 6 *mmol/l*, the GP will say that these levels are alright. [...] According to the Quattro high-risk guideline, however, these levels are too high; [...] So, the GPs also learned from this new guideline, as we *(the practice nurses) *used the guideline saying these levels are too high; we have to do something. The GPs also have learned that a cholesterol level of 6.4 *mmol/l *can be seen as a risk for patients with cardiovascular diseases" (interview practice nurse 27-01-2006). So, as the Quattro guideline was an integration of separately existing guidelines and focussed on the interdependency of risk factors, the physical outcome measures in the separately existing guidelines were altered, making a cholesterol level of 6 mmol/l too high. As the guideline provided gave the practice nurses the position to negotiate appropriate treatments with the GPs, it provided them with a more crucial role in organising this prevention programme as they all remained in the health care centres as practice nurses even after the Quattro study had ended.

## Discussion

Pragmatic trials, which have been developed since the 1980's to evaluate the effectiveness of treatments and/or complex interventions as they are used in routine practice [25,26], are seen as a way to overcome the incompatibility of general guidelines and recommendations with local knowledge about the organisation of routine medical practice and the relationship between the professionals and the patients. However, as we have shown the liberty provided by a tailor-made approach for health care professionals to develop their preferred procedures for the intervention, did not result in the organisation of multidisciplinary patient care, which was the core of the intervention. In fact, the lack of adequate guidelines hindered the implementation of the intervention.

It is often argued that guidelines coordinate medical practices [27-30], as they guide medical professionals through a sequence of steps in the management of care. As guidelines articulate and delegate tasks to professionals over different sites and time – and thus can be seen as a coordinating tool [30] – they structure an approach to diagnosis and/or treatment as a logical process ("when situation X presents itself, than Y should be done") [27,30]. So, as a guideline regulates the content and the sequence of medical work, it also regulates – to a certain extent – its organisation in practice. Guidelines in medical practice, thus, are not purely 'medical' they also involve organisational aspects. Beside having relevant clinical components, care programmes should therefore be seen as multidisciplinary protocols that encompass tasks, decision criteria and work procedures for the care professionals involved in the care trajectory of a specific patient category [31]. When every health care professional knows his/her tasks, then collaborative meetings could have relevance as formative evaluations of patients' treatment plans.

For evaluating complex interventions conventional RCTs are considered not to be appropriate, because of the rigidness of their designs, the perceived preoccupation with measuring outcomes rather than the process and the implications for health promotion practice [1-3], [32-37]. As pragmatic trials, on the other hand, measure the effectiveness of intervention under routine conditions [4,25,26,38,39], they allow for the incorporation of variations among sites, professional and patient heterogeneity, and less standardised treatments to correspond with daily clinical decision-making. In order to enable permanent uptakes of interventions numerous initiatives are deployed to incorporate these pragmatic adjustments either into the design of pragmatic trials, like phased implementation designs, formative loops, process evaluations [2,40-42] or within routine care, like community capacity building, supportive training and involving change moderators [3,43,44]. Because of this persistent emphasis on flexibility, however, we believe the pragmatic trial discourse to reflect a great fear of standardising trial designs and interventions too much. Indeed as Judd et al. [33] have argued, standardisation does have a supportive and empowering function for health care professionals in health promotion.

In other words, a tailor-made approach in pragmatic trial research should not rule out the use of guidelines or protocols. Guidelines do not standardise care practices; in practice they will always be localised and contextualised [29]. So, pragmatic trial researchers should not fear standardising care practices when applying tailor-made approaches [27,28].

## Conclusion

As tailor-made approaches are developed to enable the uptake of interventions in routine practice, they are facilitated by the brokering of tools such as guidelines. In our study, guidelines facilitated organisational change and enabled the transformation of existing interprofessional relations, and thus made tailoring possible. The attractive flexibility of pragmatic trial design in taking account of local practice variations may often be overestimated.

## Competing interests

The author(s) declare that they have no competing interests.

## Authors' contributions

YJ and AB drew up the manuscript. Ethnographic data collection on the execution of the pragmatic trial and the performance of the intervention in the health care centres was done by YJ. RB, MF, MB took part in reviewing the manuscript. All authors read and approved the final version of the article.

## Pre-publication history

The pre-publication history for this paper can be accessed here:


